# Multiple paternity in wild house mice (*Mus musculus musculus*): effects on offspring genetic diversity and body mass

**DOI:** 10.1002/ece3.920

**Published:** 2013-12-22

**Authors:** Kerstin E Thonhauser, Michaela Thoß, Kerstin Musolf, Teresa Klaus, Dustin J Penn

**Affiliations:** 1 Konrad Lorenz Institute of Ethology Department of Integrative Biology and Evolution, University of Veterinary MedicineVienna, Austria; 2 Department of Biology, Brooklyn CollegeBrooklyn, New York, USA

**Keywords:** Genetic benefits, genetic diversity, heterozygosity, house mice, multiple paternity

## Abstract

Multiple mating is common in many species, but it is unclear whether multiple paternity enhances offspring genetic diversity or fitness. We conducted a survey on wild house mice (*Mus musculus musculus)*, and we found that in 73 pregnant females, 29% of litters had multiple sires, which is remarkably similar to the 23–26% found in feral populations of *Mus musculus domesticus* in the USA and Australia, respectively. The question is: How has selection maintained multiple mating in these subspecies since the evolutionary divergence, ca. 2800–6000 years ago? We found no evidence that multiple paternity enhanced females’ litter size, contrary to the fertility assurance or genetic benefits hypotheses. Multiple paternity was associated with reduced mean and variance in offspring body mass, which suggests that females allocate fewer resources or that there is increased intrauterine conflict in multiple-versus single-sired litters. We found increased allelic diversity (though not heterozygosity) in multiple-sired litters, as predicted by the genetic diversity hypothesis. Finally, we found that the dams’ heterozygosity was correlated with the mean heterozygosity of their offspring in single-and multiple-sired litters, suggesting that outbred, heterozygous females were more likely to avoid inbreeding than inbred, homozygous females. Future studies are needed to examine how increased genetic diversity of litters and smaller mean (and variance) offspring body mass associated with multiple paternity affect offspring fitness.

## Introduction

Polyandry or multiple mating is common in diverse animal taxa ranging from insects to mammals (Arnqvist and Nilsson 2000; Wolff and Macdonald 2004), and although this behavior has been studied extensively over the past decades, there is still continuous debate over its function. Male reproductive success is usually limited by the number of mating partners acquired, whereas female reproductive success is potentially limited by the number of ova produced (Bateman 1948; Trivers 1972) or number of offspring raised. Therefore, unlike males, it is not obvious how females can increase their reproductive success by mating with multiple males. There is accumulating evidence that females actively engage in multiple mating in many species (Berteaux et al. 1999; Arnqvist and Nilsson 2000; Rolland et al. 2003; Westneat and Stewart 2003; Manser et al. 2011), despite a variety of potential costs, including an elevated risk of disease transmission, predation and injuries from potential mating partners (Daly 1978; Magnhagen 1991; Siva-Jothy 2006). These costs suggest that there are compensating benefits for mating with multiple partners, and several nonmutually exclusive hypotheses have been proposed (Jennions and Petrie 2000; Hosken and Stockley 2003). The benefits can either be direct, cryptic, or indirect (see Table 1). In nonresource-based mating systems in which males provide no parental care, explanations of polyandry largely rely on indirect or genetic benefits (Simmons 2005). Females can gain such benefits either through (i) good genes (Kempenaers et al. 1992; Keller and Reeve 1995; Yasui 1997), (ii) increased genetic compatibility (Zeh and Zeh 1997), including inbreeding avoidance (Tregenza and Wedell 2002), or (iii) enhanced genetic diversity for their offspring (Yasui 1998; Cohas et al. 2007). The good genes and compatible genes hypotheses assume that multiple mating enhances female fitness in increasing the number or quality of offspring produced (Madsen et al. 1992; Tregenza and Wedell 1998; García-González and Simmons 2005; Fisher et al. 2006), whereas the genetic diversity hypothesis assumes that females gain fitness benefits from multiple mating in producing genetically more diverse clutches (Yasui 1998). This strategy serves as a bet-hedging mechanism against unstable environments or fast-evolving parasites (Baer and Schmid-Hempel 1999) and ensures that at least some genotypes within a clutch will fit the current environmental conditions and survive. The genetic diversity hypothesis does not necessarily predict an increase in offspring number or quality if females mate with multiple mates, but that the variation in fitness among multiple-sired litters is reduced in comparison with the variation among single-sired litters (Table 1).

**Table 1:** Overview of the potential fitness benefits females can gain from polyandry and the expected consequences in a natural population of house mice.

Function	Hypothesis	Description	Expected fitness consequences	References
Direct benefits	Material benefits hypothesis	Polyandry provides females with material benefits (e.g., nuptial gifts, parental care, or other resources from males)	Female house mice are unlikely to gain material benefits from polyandry as they live in a nonresource-based mating system where males provide no parental care	Arnqvist and Nilsson (2000) Hosken and Stockley (2003)
Cryptic benefits	Convenience polyandry	Polyandry functions to avoid costs arising from rejecting multiple males as mates	MP rate is not positively correlated with litter size or weanling body mass MP rate is not positively correlated with litter genetic diversity or heterozygosity	Thornhill and Alcock (1983)
	Infanticide avoidance	Polyandry serves to conceal paternity to prevent infanticide from unmated males	MP rate is positively correlated with litter size but not with weanling body mass MP is not positively correlated with litter genetic diversity or heterozygosity	Hrdy (1979) Wolff and Macdonald (2004)
	Fertility assurance	Polyandry protects against sperm depletion or genetically incompatible males	MP rate is positively correlated with litter size but not with weanling body mass MP in not positively correlated with litter genetic diversity or heterozygosity	Hoogland (1998) Stockley (2003)
Indirect benefits	Good gene hypothesis	Polyandry provides females with intrinsic male quality which increases offspring viability	MP rate is positively correlated with litter size and weanling body mass MP rate is not positively correlated with litter genetic diversity or heterozygosity	García-González and Simmons (2005) Hosken et al. (2003)
	Genetic compatibility hypothesis	Polyandry provides females with more compatible genes (e.g., inbreeding avoidance)	MP rate is positively correlated with litter size but not weanling body mass MP rate is not positively correlated with litter genetic diversity but with offspring heterozygosity	Tregenza and Wedell (1998) Tregenza and Wedell (2002)
	Genetic diversity hypothesis	Polyandry as a bet-hedging strategy against fast-evolving parasites or unpredictable environments	MP is not positively correlated with litter size or weanling body mass Fitness variance is smaller in multiple-than single-sired litters MP rate is positively correlated with litter genetic diversity but not with offspring heterozygosity	Cohas et al. (2007) Yasui (1998)

Adapted from Wolff and Macdonald (2004) and Lane et al. (2008) to the relevance of the house mouse mating system. MP refers to multiple paternity.

In house mice, genetic paternity analyses reveal that multiple paternity is common in enclosure populations (Potts et al. 1991; Lindholm et al. 2013; Montero et al. 2013; Stockley et al. 2013) and feral populations of house mice (*M. musculus domesticus)* in the USA (Dean et al. 2006) and Australia (Firman and Simmons 2008a). In addition, behavioral observations indicate that female mice actively engage in polyandry (95% of females mated with both males when given a choice between a dominant and a subordinate male, Rolland et al. 2003). Also, comparative analyses on testis size suggest that multiple mating is common in house mice (Firman and Simmons 2008a; Soulsbury 2010). On average 25% of wild *M. musculus domesticus* litters are multiple sired, although it is unclear why there is so much variation among different populations (6–43%, Dean et al. 2006; Firman and Simmons 2008a) or whether these findings can be extrapolated to *M. musculus musculus* populations in Europe. In mice and bank voles (*Clethrionomys glareolus*), population density and the number of available males both correlate with multiple paternity (Dean et al. 2006; Klemme et al. 2007). In addition, the rate of multiple mating might show seasonal variation as food availability – an important determinant of population dynamics – varies strongly across seasons; however, this hypothesis has not before been investigated to our knowledge. Experiments under laboratory conditions revealed that female house mice gain indirect genetic benefits from polyandry. For example, postnatal pup survival was increased in females that mated with three different males in comparison with females that mated three times with the same male (Firman and Simmons 2008c). Also, polyandry facilitates inbreeding avoidance (Firman and Simmons 2008b) and polyandrous females produce sons that are superior in sperm competition (Firman 2011). However, it is unclear whether or how these findings apply to natural populations as selection is likely to be stronger in the wild and the degree of multiple mating might vary according to population demographics and environmental circumstances.

**Figure 1:**
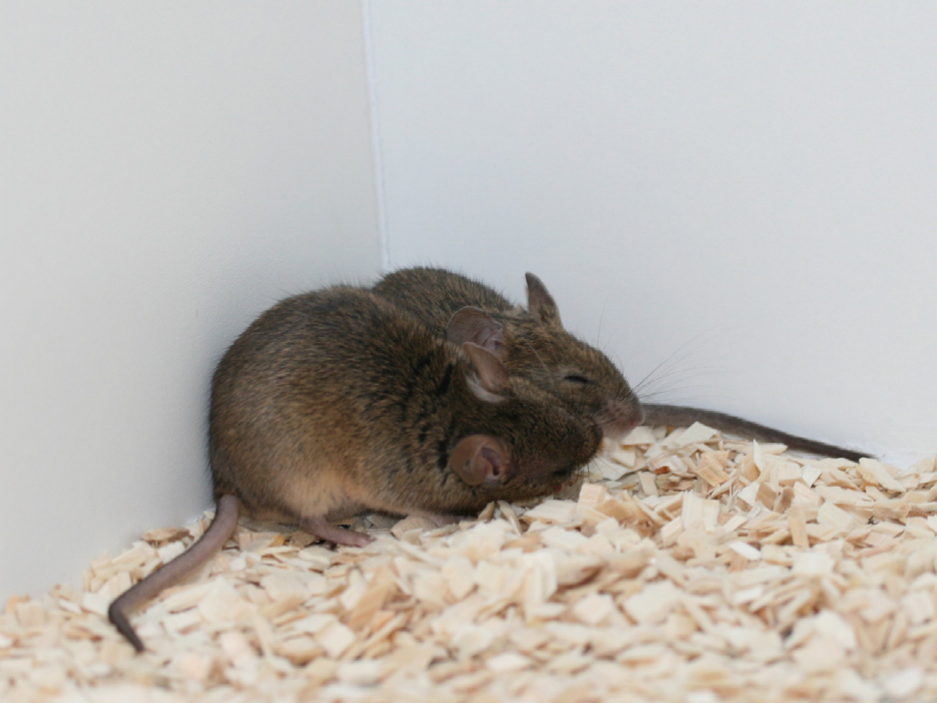
Male and female house mouse (*Mus musculus musculus*).

In this study, we investigated the frequency of multiple paternity in wild house mice (*M. musculus musculus)* (Fig. 1) and compared two distinct populations over different seasons. We examined differences in litter size, weanling body mass, male sex ratio, litter genetic diversity, and litter observed heterozygosity among multiple-and single-sired litters to assess the potential fitness benefits females gain from polyandry.

## Materials and Methods

### Animal trapping and housing

Trapping was conducted on regular intervals in the years 2004–2007 and 2010. Even though trapping was conducted throughout the year, more trapping effort was set during summer months. In total, we trapped 73 pregnant female house mice (winter *N* = 7, spring *N* = 15, and summer *N* = 51) at two different sites in and around Vienna, Austria (KLIVV: 48°12′38″N, 16°16′54″E, *N *=* *65; Safari park: 48°18′22″N, 16°43′48″E, *N *=* *8). These two sites both contained commensal populations.

For animal trapping, we used Sherman live traps. Traps were provided with food (piece of apple, peanut butter, and dry bread) and nesting material (wood shavings and cotton). Trapping was conducted either during dusk or dawn, and traps were checked for occupancy at least once during a six-hour interval. Trapped animals were returned to the colony and placed individually in Type IIL cages (Tecniplast, 32.5 × 20.5 × 14 cm). Cages contained wood shavings (ABEDD) and nesting material for environmental enrichment. Mice were kept under a 12:12-h dark/light cycle and provided with food (Altromin rodent diet 1324) and water ad libitum. Offspring were weaned at 21 ± 1 days and kept under standard colony conditions. All pups were sexed at weaning and litter size was recorded at birth. In 2010, we additionally measured offspring body mass (g) at weaning (*N *=* *30 litters). Ear punches were collected for individual identification, and tissues were stored at −20°C for subsequent genetic analyses.

### Genotyping and paternity analysis

DNA was extracted from ear punches using a proteinase K/isopropanol protocol (Sambrook et al. 1989). A total of 73 adult females and 369 offspring were genotyped at a subset of 16 microsatellite loci (D1Mit404, D1Mit456, D2Mit252, D2Mit380, D5Mit25, D6Mit138, D7Mit227, D9Mit34, D9Mit135, D10Mit20, D11Mit150, D15Mit16, D17Saha, D17Mit28; D17Mit 21, D19Mit39; see Mouse Microsatellite Data Base of Japan) using a Multiplex-PCR Master Mix (Qiagen Multiplex PCR kit, Qiagen, Venlo, Netherlands). In the years 2004–2007, females and offspring were typed for at least 10 and on average at 12 loci. In 2010, mice were typed for at least 11 and on average at 13 loci. The markers are located on 11 different chromosomes and were previously screened to confirm that they were polymorphic. The markers include three microsatellites closely linked to major histocompatibility complex (MHC): D17Saha and D17Mit21 are located within the MHC class II E *β* locus and A *α* locus, respectively (Saha and Cullen 1986; Meagher and Potts 1997), D17Mit28 is adjacent to MHC class I K locus (Dietrich et al. 1996; Meagher and Potts 1997). Amplification mixes were subjected to a denaturation step at 94°C for 15 min followed by 30 cycles at 94°C for 30 sec, 55°C for 90 sec and 72°C for 60 sec, followed by an elongation step at 72°C for 10 min. Amplification products were analyzed using an automated sequencer (Beckman Coulter CEQ 8000; Beckman Coulter, Pasadena, CA). Allele scoring was made using Beckman Coulter CEQ 8000 System software, and allele sizes were determined with SLS+400 as size standard. Estimated number of fathers per litter was obtained using the program GERUD 2.0 (Jones 2005). This program removes maternal alleles from the offspring genotypes and uses multiple loci simultaneously to simulate all possible paternal genotypes before calculating the combinations of these genotypes that yield the fewest possible number of fathers that could have contributed to the observed offspring genotypes.

### Estimating genetic diversity

Mean number of alleles per locus was calculated separately for each litter using the program FSTAT developed by Jérôme Goudet (downloadable from: http://www2.unil.ch/popgen/softwares/fstat.htm). Observed multilocus heterozygosity (number of heterozygous loci divided by the total number of genotyped loci) was calculated using IRmacroN4, a macro for Microsoft Excel written by Amos (downloadable from: http://www.zoo.cam.ac.uk/zoostaff/amos/#ComputerPrograms).

### Statistical analyses

We first tested whether population or trapping season had a significant effect on the rate of multiple paternity. Therefore, we applied a generalized linear mixed effects model (GLMM) with a binomial distribution and a logit link function using paternity (single or multiple) as the dependent variable, population and trapping season as fixed factors. We also included dam's observed heterozygosity as a covariate to assess whether dams’ heterozygosity was correlated with multiple paternity. Trapping year was set as random factor to control for the variation and nonindependence across trapping years. Second, to determine whether litter size was affected by paternity or differed over population or trapping seasons, we applied a linear mixed effects model (LMM) with litter size as dependent variable; paternity, population, and trapping season as fixed factors; and trapping year as a random factor. As the likelihood of detecting multiple paternity increases with litter size, we also used a second measure, the paternity share, which is independent of litter size. Paternity share is an estimate of the probability that a pup was sired by another male than the primary male. Paternity share was calculated using the method of Eccard and Wolf (2009). Third, to test whether litter size predicted mean weanling body mass, we ran a general linear model (LM) with mean weanling body mass as the dependent variable and litter size as a covariate. We could not test for population differences in mean weanling body mass, as offspring body mass data were only collected in the KLIVV population in 2010 (*N *=* *30 litters). To test for differences in mean weanling body mass of single-versus multiple-sired litters, we applied a Wilcoxon test. Homogeneity of variances was tested using Levene test. To determine whether multiple paternity affected offspring sex ratio, we calculated a GLMM with a binomial distribution and a logit link function with the number of male offspring as dependent variable; litter size as the binomial denominator; and paternity, population, and trapping season as fixed factors. Again we included trapping year as a random factor. Fourth, to test for differences in offspring genetic diversity, we applied a LMM with the mean number of alleles per litter as dependent variable; paternity, population, and season as fixed factors; and litter size as a covariate. We included trapping year as a random factor. Finally, to test which factors influence offspring observed heterozygosity, we ran a LMM with mean offspring heterozygosity within the litter as dependent variable; and paternity, population, and season as fixed factors. We included observed heterozygosity of the dam as a covariate into the model to test whether the dam's observed heterozygosity correlated with mean offspring heterozygosity. Again we included trapping year as a random factor. We verified that model assumptions (i.e., normally distributed residuals and homogeneity of variances) were fulfilled and log-transformed data if necessary. We applied a backward stepwise removal procedure (Grafen and Hails 2002) to avoid problems due to inclusion of nonsignificant terms (Engqvist 2005), and the removed variables were reentered one by one to the final model to obtain relevant statistics. Statistical analyses were performed using “R” (version 2.14.1) (R Development Core Team 2012). We implemented linear mixed effects models using the “lme” function of the “nlme” package (Pinheiro et al. 2012) and generalized mixed effects models using the “lmer” function in the “lme4” package (Bates et al. 2011). For post hoc analyses, we used the “glht” function of the “multcomp” package (Hothorn et al. 2008).

## Results

Overall, we found that 21 out of 73 litters had multiple sires (these litters had two sires except for one litter, which was sired by three males) revealing that the rate of multiple paternity was 29% (95% confidence interval (CI): 19.2–38.4%). The paternity share was estimated as 6.6% (95% CI: 4.2–9.2%). We found no difference in the frequency of multiple paternities between the populations (GLMM: *χ*² = 0.549, *N *=* *73, *P *=* *0.459) or between seasons (GLMM: *χ*² = 2.658, *N *=* *73, *P *=* *0.264).

**Figure 2:**
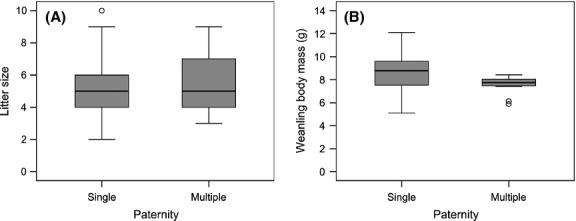
(A) Litter size of single-and multiple-sired litters and (B) mean weanling body mass (g) within single-and multiple-sired litters.

We examined whether multiple paternity was correlated with litter size and weanling body mass, and we found that multiple paternity did not affect litter size (LMM: *F*_1,64_ = 2.411, *P *=* *0.125) (Fig. 2A). We found no difference in litter size between populations (LMM: *F*_1,64_ = 0.180, *P *=* *0.673) or trapping seasons (LMM: *F*_2,64_ = 1.529, *P *=* *0.225). However, we found that mean and variance of weanling body mass within litters were significantly smaller in multiple-versus single-sired litters (Wilcoxon rank-sum test: *W *=* *153, *N *=* *30, *P *=* *0.037; Levene test: *F *=* *4.971, *N *=* *30, *P *=* *0.034) (Fig. 2B). Mean weanling body mass was not affected by litter size (LM: *F*_1,28_ = 2.209, *P *=* *0.148). We found no evidence that multiple paternity affected the sex ratio of weanlings (GLMM: *χ*² = 0.344, *N *=* *63, *P *=* *0.557). Male sex ratio did not differ between populations (GLMM: *χ*² = 0.162, *N *=* *63, *P *=* *0.687) or over trapping seasons (GLMM: *χ*² = 4.892, *N *=* *63, *P *=* *0.087).

**Figure 3:**
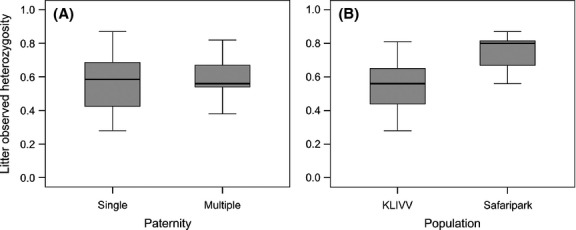
(A) Offspring mean observed heterozygosity of single-versus multiple-sired litters. (B) Offspring mean observed heterozygosity in the KLIVV and Safari park populations.

**Figure 4:**
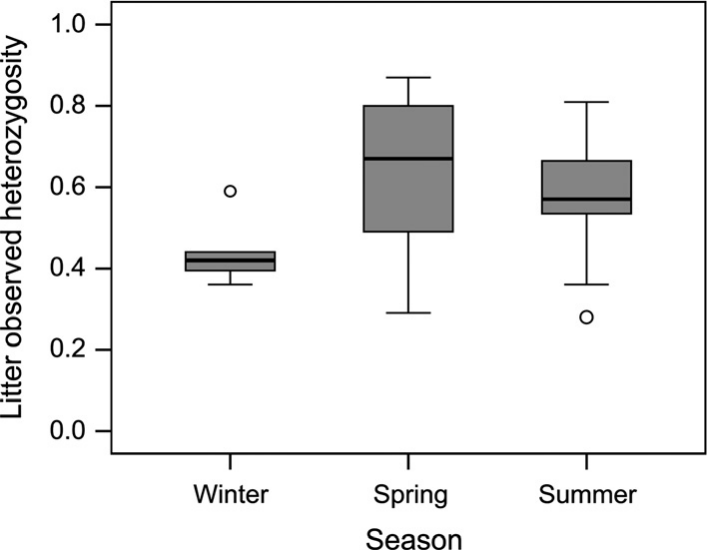
Offspring mean observed heterozygosity in litters born in winter, spring or summer. Circles refer to outliers.

**Figure 5:**
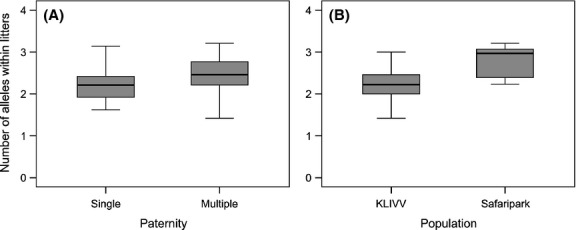
(A) Offspring mean number of alleles within single-and multiple-sired litters. (B) Offspring mean number of alleles within litters from the KLIVV or Safari park population.

We tested whether multiple paternity enhanced the genetic diversity of dams’ litters. We found no difference in the mean observed heterozygosity between single-and multiple-sired litters (LMM: *F*_1,62_ = 0.006, *P *=* *0.939) (Fig. 3A). Nonetheless, we found the mean observed heterozygosity to be significantly greater in the Safari park population (LMM: *F*_1,63_ = 11.469; *P *=* *0.001) (Fig. 3B) and a significant effect of season (LMM; *F*_2,63_ = 3.585, *P *=* *0.034): Heterozygosity was significantly lower in litters trapped in winter compared with spring and summer (winter/spring: *t *=* *2.75; *P *=* *0.020; winter/summer: *t *=* *3.389; *P *=* *0.003; spring/summer: *t *=* *0.030; *P *=* *0.999) (Fig. 4). Unlike heterozygosity, we found that the mean number of alleles was significantly higher in multiple-compared with single-sired litters (LMM: *F*_1,65_ = 4.235, *P *=* *0.044) (Fig. 5A). Litter size had no influence on the mean number of alleles within litters (LMM: *F*_1,64_=0.074, *P *=* *0.786). Also, we did not detect any seasonal differences (LMM: *F*_2,63_ = 0.319, *P *=* *0.728). However, litters from the Safari park population had a significantly higher number of alleles than litters from the KLIVV population (LMM: *F*_1,65_ = 15.582, *P *<* *0.001) (Fig. 5B). Overall, the mean number of alleles found in the two populations (KLIVV: 2.25; Safari park: 2.79) was comparable to feral populations in Australia ranging from 2.0 to 2.91 (Firman and Simmons 2008a) but lower than the average rate found in populations in the USA (5.9) (Dean et al. 2006).

**Figure 6:**
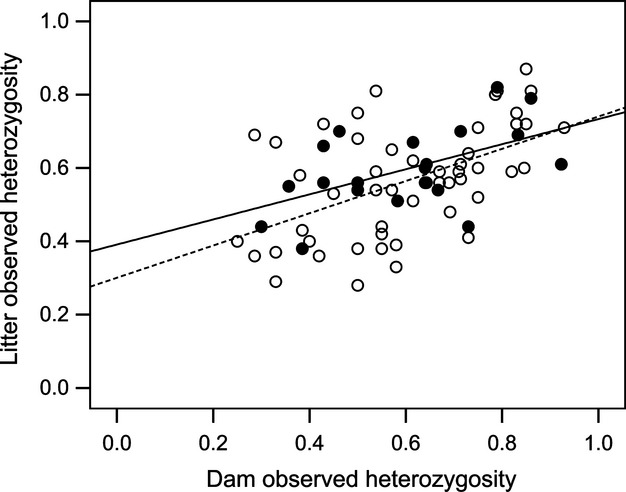
Correlation between dams' observed heterozygosity and offspring mean heterozygosity for single (white, dashed line *R*² = 0.27)-and multiple (black, solid line, *R*²* *= 0.29)-sired litters.

Finally, we were interested whether more heterozygous mothers produce more heterozygous litters than other females. We found that the dam's heterozygosity was significantly positively correlated with offspring mean heterozygosity (*F*_1,63_ = 20.695, *β *= 0.337, SE = 0.074, *P *<* *0.001) (Fig. 6). However, we found no evidence that more heterozygous females were more likely to have multiple-sired litters (GLMM: *χ² *= 2.159, *N *=* *73, *P *=* *0.142).

## Discussion

We found that multiple paternity in free ranging *M. musculus musculus* populations occurred in 29% of litters and this average rate is comparable to feral *M. musculus domesticus* populations in the USA and Australia (average rate of 23% in the USA, Dean et al. 2006; and 26% in Australia, Firman and Simmons 2008a). The rates of multiple paternity are surprisingly similar in these two subspecies – despite that they live in different continents and are reproductively isolated (2800–6000 years ago, Boursot et al. 1993) – suggesting that multiple mating is selectively maintained. Although we found that on average 29% of the litters were multiple sired, the actual rate of multiple mating might be higher depending on the competitive sire skew between males (Dean et al. 2006). A high competitive skew (one male sires the majority of offspring within a litter) requires an increased rate of multiple mating to detect multiple paternity. In house mice, observational data from the field (Firman and Simmons 2008a) and laboratory experiments (Firman and Simmons 2008c) showed that paternity is strongly biased toward one male, indicating that our measurement of multiple paternity is a conservative estimate of the rate of multiple mating. Unlike previous studies (Dean et al. 2006; Firman and Simmons 2008a), we found no difference in multiple paternity between the populations we surveyed, but we only compared two populations. The high variation in multiple paternity among populations in previous studies was suggested to be due to population density (Dean et al. 2006; but see Firman and Simmons 2008a); however, we did not find any seasonal differences in the multiple paternity rate, as would be expected if multiple paternity is density dependent (Briese and Smith 1974). Also, we did not find any seasonal effects on litter size or male sex ratio.

There are several ways how multiple paternity can affect the fitness of females and their offspring, and we examined the effects on litter size and weanling body mass. We found no difference in the litter size of single-versus multiple-sired litters, which is expected by fertility assurance and genetic benefits hypotheses. However, genetic benefits could become apparent at different life-history stages and are not restricted to greater offspring number. Unlike ground squirrels, which restrict mating to a very short time period after hibernation (Murie and Michener 1984), house mice can reproduce all year-round and do not synchronize estrus. Therefore, sperm depletion in males might be rare and unlikely to explain female multiple mating behavior in this species. Surveys of feral *M. musculus domesticus* populations in the USA and Australia found no effects of multiple paternity on litter size (embryo number in utero) (Dean et al. 2006; Firman and Simmons 2008a). Therefore, multiple paternity does not appear to increase litter size in house mice living under natural conditions. In contrast, a recent study by Firman and Simmons (2012) revealed that females kept under laboratory conditions in a polyandrous mating regime significantly increased litter size in comparison with monandrously mating females over 15 generations. This result indicates that polyandry can enhance litter size – at least under laboratory conditions without nutritional limitations. However, whether this increase in litter size is beneficial for females' fitness (lifetime reproductive success) still needs to be examined in more natural conditions.

We found that mean weanling body mass was significantly reduced in multiple-compared with single-sired litters, contrary to the hypothesis that polyandry confers genetic benefits. This reduction in offspring body mass was not due to a life-history trade-off between offspring number and quality as offspring body mass was not affected by litter size. One potential explanation is that multiple paternity is largely due to sexual coercion or infanticide avoidance, and females reduce their maternal investment when coerced into mating with nonpreferred males (Drickamer et al. 2000). Or, female weight or condition might be correlated with both their mating behavior and the body mass of pups produced. For example, small females might be more likely to mate with multiple males as they could be less effective in preventing male harassment and also produce pups with lower body mass. A nonmutually exclusive hypothesis for this result could be increased prenatal sibling rivalry in multiple-sired litters (Hager and Johnstone 2006; Hudson and Trillmich 2008). As body mass at weaning enhances offspring survival in the wild (Baker and Fowler 1992), our finding suggests that multiple paternity may have negative fitness effects for females and their offspring, which is consistent with sexual conflict hypotheses (convenience polyandry and infanticide avoidance). On the other hand, the difference in weanling weight could be attributable to sire effects. For example, if males from single-sired litters were better sperm competitors and managed to outcompete all other rivals, theory suggests that they might also sire better quality offspring (Yasui 1997). Therefore, we cannot exclude that even in single-sired litters, females might have benefitted from multiple mating. Although we found no evidence that multiple paternity increased observed heterozygosity of litters, we found that multiple-sired litters showed increased genetic diversity as the number of alleles was significantly higher in multiple-versus single-sired litters. Similar findings have been made in the alpine marmot (*Marmota marmota*) (Cohas et al. 2007), which contradicts suggestions that multiple paternity cannot increase offspring genetic diversity (Williams 1975). Increased genetic diversity of litters can enhance female fitness through bet-hedging (Yasui 1998), especially because gene-by-environment interactions on fitness are widespread (Narraway et al. 2010). Our finding that multiple-sired litters show reduced variance in offspring body mass is consistent with the bet-hedging hypothesis (assuming that body mass is a good indicator of fitness, see Baker and Fowler 1992). By reducing either the individual variance in fitness or fitness correlations between individuals from the same genetic lineage, bet-hedging could favor multiple paternity even despite a reduction in arithmetic mean fitness (Philippi and Seger 1989; Starrfelt and Kokko 2012). However, increased genetic diversity of litters might provide females with only small or even negligible fitness benefits, as it could be a nonadaptive byproduct of multiple mating, which is selectively maintained for other reasons, such as avoiding infanticide or harassment.

Inbreeding can have negative fitness consequences in house mice (Meagher et al. 2000; Ilmonen et al. 2008), and if multiple mating functions to facilitate inbreeding avoidance, one might expect higher rates of multiple paternity in inbred populations with reduced genetic variation. We found a significant difference in the genetic diversity between the two observed populations (both the number of alleles and the observed heterozygosity within litters were significantly higher in the Safari park versus KLIVV population); however, we found no difference in the rate of multiple paternity between these populations and multiple-sired litters did not show increased heterozygosity. Nonetheless, these results do not rule out the hypothesis that multiple mating functions to reduce inbreeding, especially because we only examined two populations and the genetic differences between these populations were small. Also, the comparatively small sample size in one of the populations does not allow strong inferences on this negative result. We found that offspring observed heterozygosity was significantly lower in winter compared with spring or summer, suggesting that these populations showed increased levels of inbreeding during winter, but we did not find any evidence that multiple paternity was higher in winter compared with spring or summer.

Finally, we found that dam and offspring heterozygosity were positively correlated, and this relationship was significant in both single-and multiple-sired litters. Heterozygosity can increase disease resistance (Coltman et al. 1999; Reid et al. 2005; Charpentier et al. 2008; Ilmonen et al. 2008), survival (Richardson et al. 2004) and reproductive success (Foerster et al. 2003; Kempenaers 2007), suggesting that females obtain indirect benefits by producing heterozygous offspring. The idea that heterozygosity can be heritable is controversial, but there is evidence to support this idea from some other studies (Mitton et al. 1993; Bensch et al. 2006; Hoffman et al. 2007). The most likely explanation for our finding is that heterozygous females are more likely to avoid inbreeding than inbred females (either through pre-or postcopulatory mechanisms, including dispersal), and future studies are needed to test this hypothesis.
